# Direct and acute effects of advanced glycation end products on proteostasis in isolated mouse skeletal muscle

**DOI:** 10.14814/phy2.16121

**Published:** 2024-06-19

**Authors:** Haiyu Zhao, Ryota Iyama, Eriko Kurogi, Tatsuya Hayashi, Tatsuro Egawa

**Affiliations:** ^1^ Laboratory of Sports and Exercise Medicine, Graduate School of Human and Environmental Studies Kyoto University Kyoto Japan; ^2^ Laboratory of Molecular Exercise Adaptation Sciences, Graduate School of Human and Environmental Studies Kyoto University Kyoto Japan

**Keywords:** AGEs, ER stress, protein synthesis, skeletal muscle

## Abstract

Advanced glycation end products (AGEs) have been implicated in several skeletal muscle dysfunctions. However, whether the adverse effects of AGEs on skeletal muscle are because of their direct action on the skeletal muscle tissue is unclear. Therefore, this study aimed to investigate the direct and acute effects of AGEs on skeletal muscle using an isolated mouse skeletal muscle to eliminate several confounders derived from other organs. The results showed that the incubation of isolated mouse skeletal muscle with AGEs (1 mg/mL) for 2–6 h suppressed protein synthesis and the mechanistic target of rapamycin signaling pathway. Furthermore, AGEs showed potential inhibitory effects on protein degradation pathways, including autophagy and the ubiquitin–proteasome system. Additionally, AGEs stimulated endoplasmic reticulum (ER) stress by modulating the activating transcription factor 6, PKR‐like ER kinase, C/EBP homologous protein, and altered inflammatory cytokine expression. AGEs also stimulated receptor for AGEs (RAGE)‐associated signaling molecules, including mitogen‐activated protein kinases. These findings suggest that AGEs have direct and acute effect on skeletal muscle and disturb proteostasis by modulating intracellular pathways such as RAGE signaling, protein synthesis, proteolysis, ER stress, and inflammatory cytokines.

## 1 INTRODUCTION

The skeletal muscle is a major organ of biological activity and is considered a significant constituent organ of body metabolism. However, along with age, the skeletal muscle can experience atrophy and muscle regeneration failure, thereby leading to loss of mobility (Mitchell et al., 2012). This is known as sarcopenia and is mainly characterized by a decrease in the skeletal muscle cross‐sectional area and the number of muscle fibers, with loss of muscle strength (Wilkinson et al., 2018).

Advanced glycation end products (AGEs) are derived from the non‐enzymatic glycation of proteins and primarily reducing sugars (Vistoli et al., 2013). There are two main sources of AGEs accumulated in the human body; one is AGEs synthesis in the body, and the other is the intake of AGEs present in food into the body (Nie et al., 2022). AGEs accumulate with age in serum (Uribarri et al., 2007) and in other sites, including the skin (Momma et al., 2011) and muscles (Olson et al., 2021). Several pieces of evidence have stated that AGEs have become one of the significant causes of age‐related diseases, including type 2 diabetes (Khalid et al., 2022), Alzheimer's disease (Takeuchi & Yamagishi, 2008), and cancer (Muthyalaiah et al., 2021). Therefore, in recent years, AGEs have been widely noticed in relevant studies, including age‐related diseases.

The balance between protein synthesis and degradation in the skeletal muscle is one of the main factors affecting sarcopenia (Nakao et al., 2019). The mechanistic target of rapamycin (mTOR) signaling pathway is vital for protein synthesis. mTOR phosphorylation activates protein synthesis by phosphorylating 70 kDa ribosomal protein S6 kinase (p70S6K), which is downstream of the mTOR pathway (Mascher et al., 2011; Wang et al., 2001). Our laboratory has previously reported reduced skeletal muscle weight and reduced muscle strength in mice exposed to AGEs for 16 weeks and reduced p70S6K activation of the protein synthesis pathway (Egawa et al., 2017). Furthermore, in our previous study on C2C12 skeletal muscle cells, the phosphorylation of mTOR, p70S6K, and 4E binding protein 1 (4E‐BP1) in the insulin pathway was reduced by 24 h AGEs exposure (Egawa et al., 2018). Moreover, another study has shown that AGEs induce skeletal muscle atrophy by modulating the Akt pathway in cultured muscle cells (Chiu et al., 2016). These results suggest changes in AGE‐induced intracellular pathways that affect the normal proteostasis of the skeletal muscle.

Some AGEs produced in the body and AGEs taken into the body from the diet bind to receptors for AGEs (RAGE), which are mainly located on the cell membrane, affecting the intracellular pathway changes (Khalid et al., 2022; Muthyalaiah et al., 2021). The binding of AGEs to RAGE has been suggested to induce skeletal muscle dysfunction through mitochondrial dysfunction, promoting inflammation, and altering cellular proteostasis (Daussin et al., 2021). Our recent study has shown that the AGEs–RAGE axis promotes disuse muscle atrophy by inducing protein degradation and inflammatory cytokine production (Egawa et al., 2020). Furthermore, the AGEs–RAGE axis induces endoplasmic reticulum (ER) stress in skeletal muscle cells and stimulates unfolded protein response (UPR) by activating the PKR‐like ER kinase (PERK) (Du et al., 2023). Another study reported that AGE‐stimulated aortic endothelial cell proliferation was reduced, and C/EBP homologous protein (CHOP), a downstream target of the UPR, was induced to promote apoptotic cell death (Adamopoulos et al., 2014). Regarding mitochondrial function, the AGEs–RAGE axis has been shown to impair mitochondrial function in the soleus muscle by reducing the respiratory chain complex activity (Velayoudom‐Cephise et al., 2020).

A significant limitation of the previous studies, however, is that it is unclear whether AGEs directly act on the skeletal muscle tissue to cause the abovementioned responses or whether they are induced acutely by exposure over hours rather than days or months. Clarifying this point is important for understanding the exact role of AGEs in skeletal muscle. To clarify these important limitations, in this study we aimed to clarify the direct and acute effect of AGEs on intracellular pathways, including protein synthesis, protein degradation, ER stress, mitochondrial proteins, and inflammatory cytokines. We hypothesized that AGEs stimulation can directly and acutely affect proteostasis in skeletal muscle tissue through the above‐mentioned pathways. For this purpose, we used isolated mouse skeletal muscle preparations, which allowed us to analyze the direct and acute impact of AGEs on skeletal muscle by eliminating the effects of systemic confounders such as circulatory, humoral, and neurological factors, as well as intestinal absorption of AGEs.

## 2 MATERIALS AND METHODS

### 2.1 AGEs preparation

AGEs were prepared as previously described (Suzuki et al., 2023). In brief, 50 mg/mL of bovine serum albumin (BSA: SIGMA‐ALDRICH, St. Louis, A7906, MO, USA) was incubated at 37°C for 1 week with 0.1‐M glyceraldehyde (SIGMA‐ALDRICH, 49800) in phosphate buffer. As a control, BSA without glyceraldehyde was prepared under the same conditions. The unreacted glyceraldehyde and excess ions are subsequently dialyzed using Slide‐ALyzer Dialysis Cassette G2 (20K MWCO, 70 mL, Thermo Fisher Scientific, 87,738, Waltham, MA, USA) against phosphate‐buffered saline. We confirmed that the AGEs solution prepared using this method contains 36 times more fluorescent AGEs than the BSA solution (Egawa et al., 2024).

### 2.2 Isolation of skeletal muscle and incubation

Twenty‐seven 10‐week‐old male C57Bl/6NCr mice were purchased from Shimizu Breeding Laboratories (Kyoto, Japan). All animal protocols were performed in accordance with the Guide for the Care and Use of Laboratory Animals by the National Institutes of Health (Bethesda, MD, USA) and were approved by the Kyoto University Graduate School of Human and Environmental Studies (approval number: 21‐A‐1).

Mice were fasted overnight before experiments (from 18:00 pm). Mice were killed by cervical dislocation without anesthesia, and extensor digitorum longus (EDL) muscles were dissected from the left and right hind limbs of mice. We chose EDL because fast‐type muscles are more susceptible to glycative stress than slow‐type muscles (Egawa et al., 2017; Egawa et al., 2019). Both ends of each muscle were secured with sutures, and the muscles were mounted on an incubation apparatus with tension at resting length. Muscles were preincubated for 30 min in 7‐mL Krebs–Ringer bicarbonate buffer (117 mM NaCl, 4.7 mM KCl, 2.5 mM CaCl_2_, 1.2 mM KH_2_PO_4_, 1.2 mM MgSO_4_, 24.6 mM NaHCO_3_) containing 2‐mM sodium pyruvate and 0.005% antiform. During incubation, buffers were continuously gassed with 95% O_2_–5% CO_2_ and maintained at 37°C. Muscle resting tension was set to 0.5 g following a previous study (Fujii et al., 2005).

Subsequently, the muscle tissues were randomly incubated with fresh buffer containing 1 mg/mL of AGEs or BSA. We determined the AGEs stimulation concentration and time based on previous studies (Mahali et al., 2014; Pinto et al., 2018). For the time‐dependent effects of AGEs, muscle tissues were incubated for 2 h (AGEs, *n* = 13; BSA, *n* = 13) or 6 h (AGEs, *n* = 15; BSA, *n* = 13). For samples incubated for 6 h, the buffer was replaced with a fresh one following 3‐h incubation. The muscle tissues were subsequently frozen in liquid nitrogen for subsequent analysis.

### 2.3 Sample preparation and western blotting

Muscle samples for western blotting were prepared as previously described (Egawa et al., 2017). Briefly, muscles were homogenized by beads homogenizer (Cell Destroyer PS2000, Bio Medical Science Inc., Tokyo, Japan) in ice‐cold lysis buffer containing 20‐mM Tris–HCl (pH, 7.4), 50‐mM NaCl, 50‐mM NaF, 5‐mM Na_4_P_2_O_7_・10 H_2_O, 250‐mM sucrose, 1% Triton X‐100, and centrifuged at 16,000 × *g* for 30 min at 4°C. Then, the supernatants were collected, and the protein content was determined using Protein Assay CBB Solution (Nacalai Tesque, 11,617–71, Kyoto, Japan). The supernatants were mixed in Laemmli sample buffer and boiled at 95°C for 5 min. The samples (10 μg of protein) were separated by sodium dodecyl sulfate polyacrylamide gel and transferred to Poly Vinyli dene Fluoride (PVDF) membranes (pore size: 0.45 μm, Millipore, IPVH00010, Burlington, MA, USA). PVDF membranes were blocked using EveryBlot blocking buffer (BIO‐RAD, Hercules, CA, USA) and incubated with primary antibodies in Can Get Signal Solution 1 (Toyobo, NKB‐301, Osaka, Japan). The following are the data of the primary antibody: puromycin (Millipore, MABE343), phospho‐p70S6K (Cell Signaling Technology [CST], Danvers, MA, USA, 9234), p70S6K (CST, 9202), phospho‐mTOR (CST, 2971), mTOR (CST, 2972), phospho‐4E‐BP1 (CST, 9459), 4E‐BP1 (CST, 9452), p62 (CST, 5114), LC3B (CST, 2775), MuRF (Santa Cruz, Santa Cruz, CA, USA, sc‐398,609), Atrogin‐1 (Santa Cruz, sc‐27,645), CHOP (CST, 2895), ATF6 (Gene Tex, Arling‐ton, TX, USA, GTX104820), IRE1α (CST, 3924), phospho‐IRE1 (Abcam, ab48187), PERK (CST, 5683), phospho‐PERK (ABclonal, Woburn, MA, USA AP0886), XBP‐1 s (CST, 40435), OXPHOS (Abcam, Cambridge, UK, ab110413), PGC‐1α (Abcam, ab54481), p38MAPK (CST, 9212), phospho‐p38 MAPK (CST, 9211), SAP/JNK (CST, 9252), phospho‐SAP/JNK (CST, 9251), p44/42 MAPK (Erk1/2) (CST, 9102), and phospho‐p44/42 MAPK (Erk1/2) (CST, 9101). Subsequently, membranes were washed with TBS‐T and incubated with secondary antibodies: anti‐rabbit IgG (CST, 7074) or anti‐mouse IgG (CST, 7076) for 1 h at 25°C. Protein bands were visualized using the Chemi Lumi One (Nacalai Tesque, 11644‐40) by WSE‐6100 LuminoGraph (ATTO, Tokyo, Japan). Equal protein loading and transfer efficiency was verified by Coomassie Brilliant Blue (CBB) staining of the membranes. The signal intensity of target protein was normalized to total protein (CBB staining intensity).

### 2.4 Muscle protein synthesis measurement

In the last 30 min of incubation, the muscle tissues were replaced with a fresh buffer with 1‐μM puromycin added to each stimulation buffer to measure protein synthesis using the surface sensing of translation (SUnSET) method (Schmidt et al., 2009). Following homogenization and centrifugation at 16,000 × *g* for 30 min at 4°C, supernatants were collected and processed for western blot analysis. A mouse monoclonal anti‐puromycin antibody (MABE343, Millipore, Cambridge, MA, USA) was used for detecting puromycin incorporation, which was quantified as the sum of all protein band intensities in the western blot analysis.

### 2.5 Real‐time polymerase chain reaction (PCR)

The mRNA levels of genes were quantified using real‐time RT‐PCR analysis as previously described (Egawa et al., 2017). Total RNA was extracted using the QIAzol Lysis Reagent (QI‐AGEN Sciences, 20,874, Ven, NLD), and RNA concentration was measured using NanoDrop Lite (Thermo Scientific, Wal, MA, USA). RNA was treated with DNase I and transcribed into cDNA by PrimeScript™ RT reagent Kit with gDNA Eraser (Takara Bio, RR047A, Kusatsu, Japan). Synthesized cDNA was subsequently subjected to real‐time RT‐PCR using the Applied Biosystems StepOne (Applied Biosystems, Foster City, CA, USA) with TB Green Premix Ex Taq II (Takara Bio, RR830B, Japan). All PCR cycles had the following conditions: initial denaturation at 95°C for 30 s followed by 40 cycles of 95°C for 5 s; 60°C, 30 s with specific primers purchased from Takara Bio (Table 1).

**TABLE 1:** Specific primers used in real‐time PCR.

Gene	Forword	Reverse
IL‐6	5'CCACTTCACAAGTCGGAGGCTTA	5'TGCAAGTGCATCATCGTTGTTC
IL‐15	5'CCAGTTGCAGAGTTGGACGAAG	5'GAGGGCCATGTGTCAAGGTG
IL‐1β	5'TCCAGGATGAGGACATGAGCAC	5'GAACGTCACACACCAGCAGGTTA
TNFα	5'ACTCCAGGCGGTGCCTATGT	5'GTGAGGGTCTGGGCCATAGAA

### 2.6 Statistical analysis

Data were expressed as means ± standard deviations. All data were subjected to two‐way analysis of variance (ANOVA), and the simple main effect of data in each group was analyzed using Tukey's post‐hoc test. Statistical analyses were performed using BellCurve for Excel software (Social Survey Research Information, Tokyo, Japan). Differences between experimental results were considered statistically significant when *p* < 0.05. The effect size (Hedge's *g*) was also calculated to examine whether the effect was meaningful and practically important. We interpreted the magnitude of the effect size by using conventional thresholds of 0.2 as a smallest effect, 0.5 as a moderate effect, and 0.8 as a large effect.

## 3 RESULTS

### 3.1 AGEs inhibited protein synthesis

**FIGURE 1:**
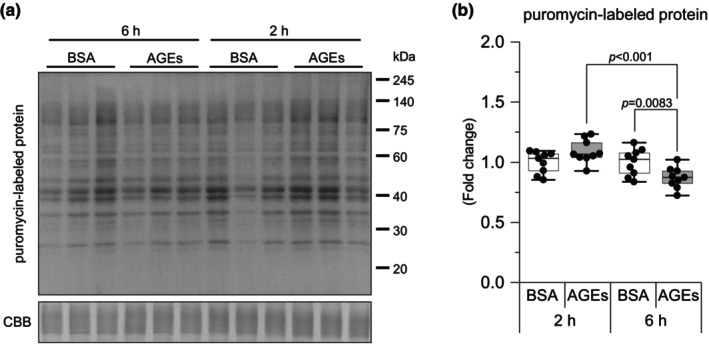
AGEs stimulation reduces protein synthesis. Protein synthesis is measured using the SUnSET method through western blot analysis. (a) Representative immunoblots from western blotting. (b) Puromycin‐labeled protein level. Data are shown as box plots. Individual data points are indicated on the graph. Statistical significance is analyzed using two‐way ANOVA with simple effects tests. *n* = 9/group.

Using the SUnSET method, we examined the effect of AGEs on protein synthesis. It was observed that compared with BSA stimulation, AGEs stimulation significantly decreased puromycin‐labeled protein level in the EDL muscle at 6 h (*p* = 0.0083, Hedge's *g* = 1.29; Figure 1b). Furthermore, compared with 2 h of AGEs stimulation, 6 h of AGEs stimulation significantly decreased puromycin‐labeled protein level in the EDL muscle (*p* < 0.001, *g* = 2.36; Figure 1b). These results suggest the suppressive effect of AGEs on protein synthesis in the skeletal muscle.

### 3.2 AGEs suppressed the activity of protein synthesis signaling

**FIGURE 2:**
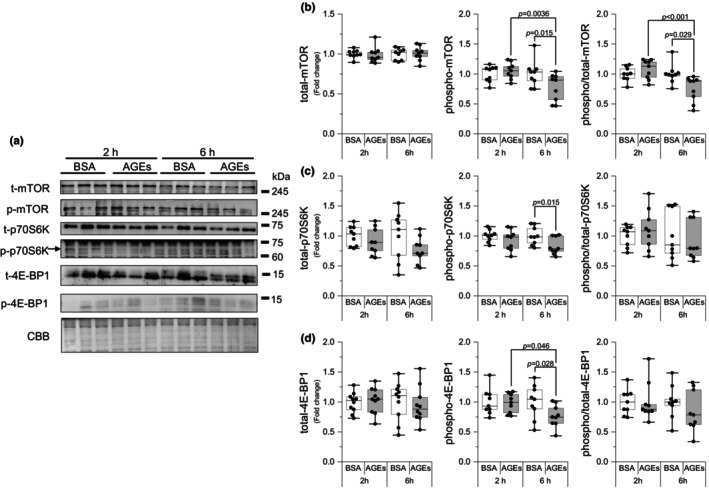
AGEs stimulation reduces the protein synthesis pathway. (a) Representative immunoblots from western blotting. (b) Total mechanistic target of rapamycin (mTOR), phosphorylation of mTOR Ser^2448^, the ratio of phosphorylation to total mTOR (6 h of AGEs vs. 6 h of BSA, *g* = 1.34; vs. 2 h of AGEs, *g* = 1.75). (c) Total 70 kDa ribosomal protein S6 kinase (p70S6K), phosphorylation of p70S6K Thr^389^, the ratio of phosphorylation to total p70S6K. (d) Total 4E binding protein 1 (4E‐BP1), phosphorylation of 4E‐BP1 Thr^37/46^, the ratio of phosphorylation to total 4E‐BP1. Data are shown as box plots. Individual data points are indicated on the graph. Statistical significance is analyzed using two‐way ANOVA with simple effects tests. *n* = 9/group.

Based on reduced protein synthesis, we investigated molecules associated with the mTOR pathway. This pathway is believed to be involved in protein synthesis. At 6 h, AGEs stimulation was observed to significantly reduce mTOR Ser^2448^ phosphorylation compared with BSA stimulation (*p* = 0.015, *g* = 1.00) but not at 2 h; additionally, 6 h of AGEs stimulation significantly reduced mTOR Ser^2448^ phosphorylation compared with 2 h of AGEs stimulation (*p* = 0.0036, *g* = 1.47; Figure 2b). Similarly, AGEs stimulation was observed to significantly decrease the ratio of phosphorylation to total (Figure 2b). The phosphorylation of p70S6K Thr^389^ (*p* = 0.015, *g* = 1.19, Figure 2c) and 4E‐BP1 Thr^37/46^ (*p* = 0.028, *g* = 0.98; Figure 2d), which are downstream signaling molecules of mTOR, was also observed to be significantly reduced by 6 h of AGEs stimulation. Furthermore, 6 h of AGEs stimulation significantly reduced 4E‐BP1 Thr^37/46^ phosphorylation compared with 2 h of AGEs stimulation (*p* = 0.046, *g* = 0.62; Figure 2d). There was no change in the total level of mTOR, p70S6K, and 4E‐BP1 (Figure 2b–d), while the ratio of phosphorylation to total levels of p70S6K and 4E‐BP1 was not significantly different (Figure 2c,d). These results suggest that AGEs influence protein synthesis via the mTOR pathway in the skeletal muscle.

### 3.3 AGEs stimulation activated RAGE signaling

**FIGURE 3:**
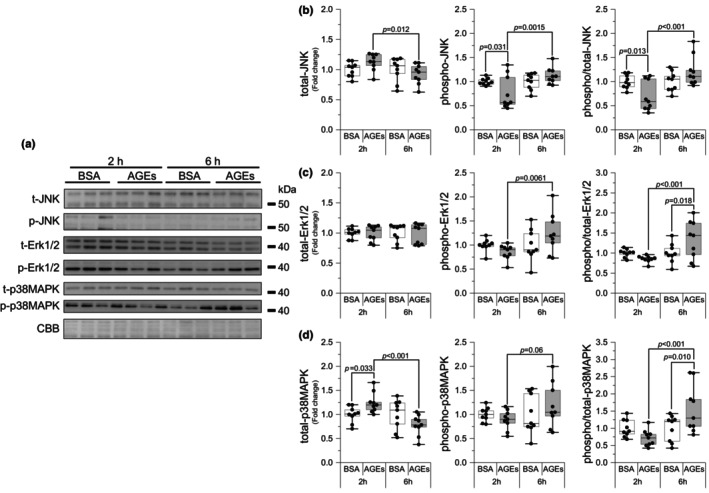
AGEs stimulate RAGE signaling. (a) Representative immunoblots from western blotting. (b) Total Jun amino terminal kinase (JNK), phosphorylation of JNK Thr^183^ / Tyr^185^, the ratio of phosphorylation to total JNK (2 h of AGEs vs. 6 h AGEs, *g* = 1.71; vs. 2 h of BSA, *g* = 1.29). (c) Total p44/42 MAPK (Erk1/2), phosphorylation of Erk1/2 Thr^202^/Tyr^204^, the ratio of phosphorylation to total Erk1/2 (6 h of AGEs vs. 2 h AGEs, *g* = 1.38). (d) Total p38 mitogen‐activated protein kinase (p38MAPK) (2 h of AGEs vs. 2 h of BSA, *g* = 1.26), phosphorylation of p38MAPK Thr^180^/Tyr^182^, the ratio of phosphorylation to total P38MAPK (6 h of AGEs vs. 2 h AGEs, *g* = 1.62). Data are shown as box plots. Individual data points are indicated on the graph. Statistical significance is analyzed using two‐way ANOVA with simple effects tests. *n* = 9/group.

The AGEs bind to RAGE to influence intracellular molecules; hence, we investigated the common RAGE signaling, MAPK signaling. Although the 2 h of AGEs stimulation decreased JNK Thr^183^/Tyr^185^ phosphorylation compared with BSA stimulation (*p* = 0.031, *g* = 0.90), it recovered after 6 h (*p* = 0.0015, *g* = 1.27; Figure 3b). The total JNK level was decreased by 6 h of AGEs stimulation (*p* = 0.012, *g* = 1.29; Figure 3b). At 6 h, AGEs stimulation significantly increased p44/42 MAPK (Erk1/2) Thr^202^/Tyr^204^ phosphorylation compared with 2 h of AGEs stimulation (*p* = 0.006, *g* = 1.25; Figure 3c). There was no change in total Erk1/2 levels, and the ratio of phosphorylation to total also increased with 6 h of AGEs stimulation compared with BSA (*p* = 0.018, g = 0.85; Figure 3c). p38MAPK Thr^180^/Tyr^182^ phosphorylation was tended to be increased by 6 h of AGEs stimulation compared with 2 h of AGEs stimulation (*p* = 0.06, *g* = 0.85; Figure 3d). AGEs simulation for 6 h decreased the total p38MAPK level (*p* < 0.001, *g* = 2.17) and the ratio of phosphorylation to total level was increased by 6 h of AGEs stimulation compared with BSA stimulation (*p* = 0.010, *g* = 0.99; Figure 3d). These results suggest that AGEs activate the RAGE signaling.

### 3.4 AGEs have the potential to reduce protein degradation pathway

**FIGURE 4:**
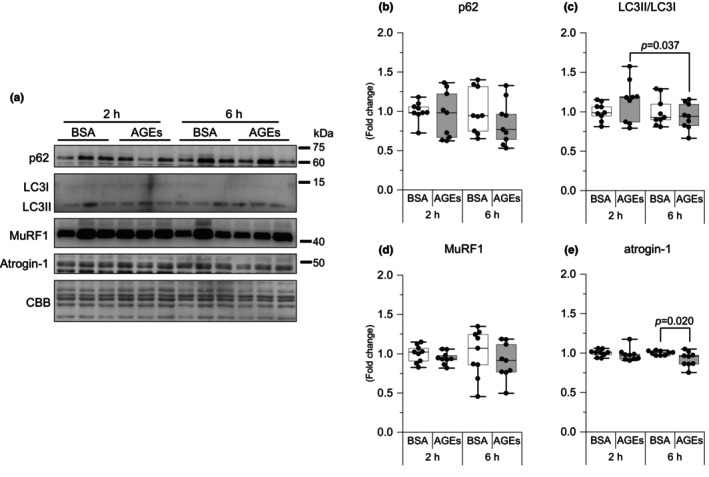
AGEs stimulation reduces the proteolysis pathway. (a) Representative immunoblots from western blotting. (b) p62. (c) Ratio of Microtubule‐associated protein 1 light chain 3 (LC3) II to LC3I. (d) Muscle RING‐finger Protein 1 (MuRF1). (e) Atrogin‐1/Muscle Atrophy F‐box (Atrogin‐1). Data are shown as box plots. Individual data points are indicated on the graph. Statistical significance is analyzed using two‐way ANOVA with simple effects tests. *n* = 9/group.

Skeletal muscle mass is controlled by the combination of protein synthesis and proteolysis. Therefore, we investigated the factors involved in protein degradation pathways. We investigated molecules, including autophagy markers, p62 and microtubule‐associated protein light chain 3 (LC3) and ubiquitin E3 ligases, muscle RING‐finger protein 1 (MuRF1), and atrogin‐1. Compared with 2 h of AGEs stimulation, LC3II/LC3I were reduced at 6 h of AGEs stimulation (*p* = 0.037, *g* = 0.35; Figure 4c). It was observed that AGEs significantly suppressed atrogin‐1 at 6 h compared with BSA but not at 2 h (*p* = 0.020, *g* = 1.12; Figure 4e). However, no changes were noted in p62 and MuRF1 (Figure 4b,d). These results suggest that AGEs have the potential to inhibit protein degradation pathways.

### 3.5 AGEs activated ER stress

**FIGURE 5:**
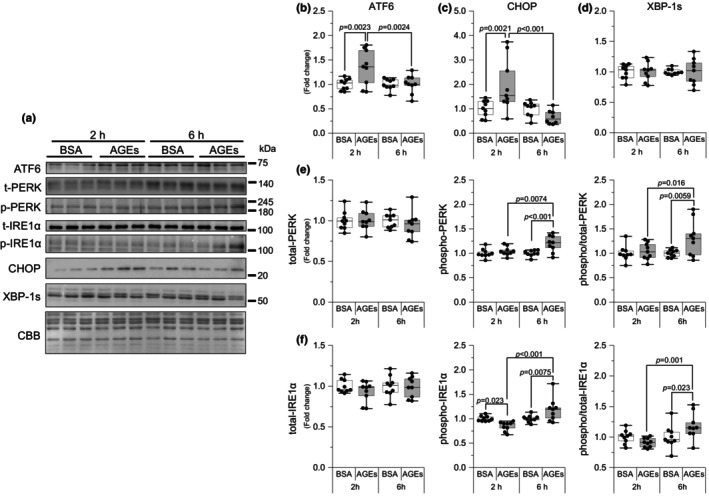
AGEs stimulate ER stress activation. (a) Representative immunoblots from western blotting. (b) Activating transcription factor 6 (ATF6). (c) C/EBP homologous protein (CHOP). (d) X‐box binding protein 1 spliced (XBP‐1 s). (e) Total protein kinase RNA‐like endoplasmic reticulum kinase (PERK), phosphorylation of PERK Thr^982^, the ratio of phosphorylation to total PERK (6 h of AGEs vs. 6 h of BSA, *g* = 1.16; vs. 2 h of AGEs, *g* = 0.93). (f) Total inositol‐requiring transmembrane kinase/endoribonuclease 1α (IRE1α), phosphorylation of IRE1α Ser^724^ (6 h of AGEs vs. 2 h of AGEs, *g* = 1.86), the ratio of phosphorylation to total IRE1α (6 h of AGEs vs. 6 h of BSA, *g* = 0.88; vs. 2 h of AGEs, *g* = 1.67). Data are shown as box plots. Statistical significance is analyzed using two‐way ANOVA with simple effects tests. *n* = 9/group.

We next examined whether AGEs have an effect on ER stress. As demonstrated in Figure 5b, 2 h of AGEs stimulation significantly increased activating transcription factor 6 (ATF6), a sensing protein of ER stress, compared with 2 h of BSA (*p* = 0.0023, *g* = 1.27) and 6 h of AGEs (*p* = 0.0024, *g* = 1.18) stimulation. At 6 h, AGEs stimulation was observed to significantly increase PERK Thr^982^ phosphorylation compared with 6 h of BSA (*p* < 0.001, *g* = 1.51) and 2 h of AGEs stimulation (*p* = 0.0074, *g* = 1.10; Figure 5e). At 6 h, AGEs stimulation was observed to significantly increase IRE1 Ser^724^ phosphorylation compared with 6 h of BSA (*p* = 0.0075, *g* = 1.04), but at 2 h, AGEs stimulation was observed to significantly reduce IRE1 Ser^724^ phosphorylation compared with 2 h of BSA (*p* = 0.023, *g* = 1.99, Figure 5f) without changes in the spliced form of X‐box binding protein 1 (XBP1s) (Figure 5d). The total protein level of PERK and IRE1 did not change, and the ratio exhibited the same change trend. As shown in Figure 5c, CHOP was increased at 2 h of AGEs stimulation compared with 2 h of BSA stimulation (*p* = 0.0021, *g* = 1.20), and the increase was reversed at 6 h of AGEs stimulation (*p* < 0.001, *g* = 1.62). These results suggest that AGEs can activate the ER stress.

### 3.6 AGEs stimulation increased mitochondria proteins

**FIGURE 6:**
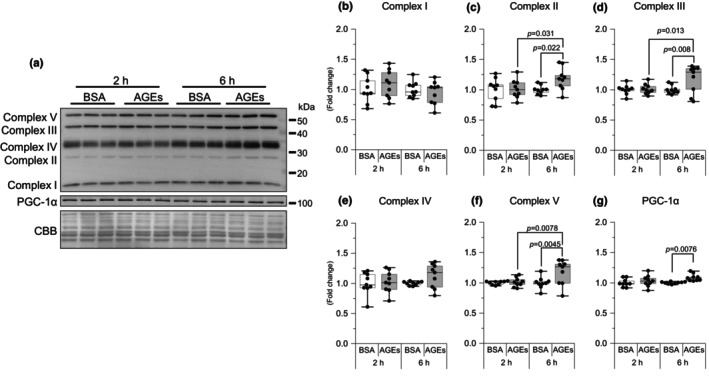
AGEs increased mitochondrial proteins. (a) Representative immunoblots from western blotting. Representative immunoblots are also shown. (b–f) Complex of oxidative phosphorylation (complexes I, II (6 h of AGEs vs. 2 h of AGEs, *g* = 0.99), III (6 h of AGEs vs. 2 h of AGEs, *g* = 0.96), IV, and V (6 h of AGEs vs. 2 h of AGEs, *g* = 1.04)). (g) Peroxisome proliferator‐activated receptor gamma coactivator 1‐alpha (PGC‐1α). Data are shown as box plots. Individual data points are indicated on the graph. Statistical significance is analyzed using two‐way ANOVA with simple effects tests. *n* = 9/group.

To determine the effect of AGEs on mitochondrial proteins, we examined the expression of mitochondria OXPHOS proteins and PGC‐1α. Exposure to AGEs for 6 h significantly increased the complex II (*p* = 0.022, *g* = 1.28; Figure 6c), complex III (*p* = 0.008, *g* = 1.05; Figure 6d), and complex V (*p* = 0.0045, *g* = 1.08; Figure 6f) of OXPHOS proteins compared with exposure to 6 h of BSA stimulation. Similar results were also obtained when compared with 2 h of BSA stimulation (Figure 6c,d,f). At 6 h of AGEs stimulation, PGC‐1α was significantly increased compared with 6 h of BSA stimulation (*p* = 0.0076, *g* = 2.03, Figure 6g). These results suggest that AGEs increase mitochondrial proteins.

### 3.7 AGEs may affect inflammatory cytokine production

**FIGURE 7:**
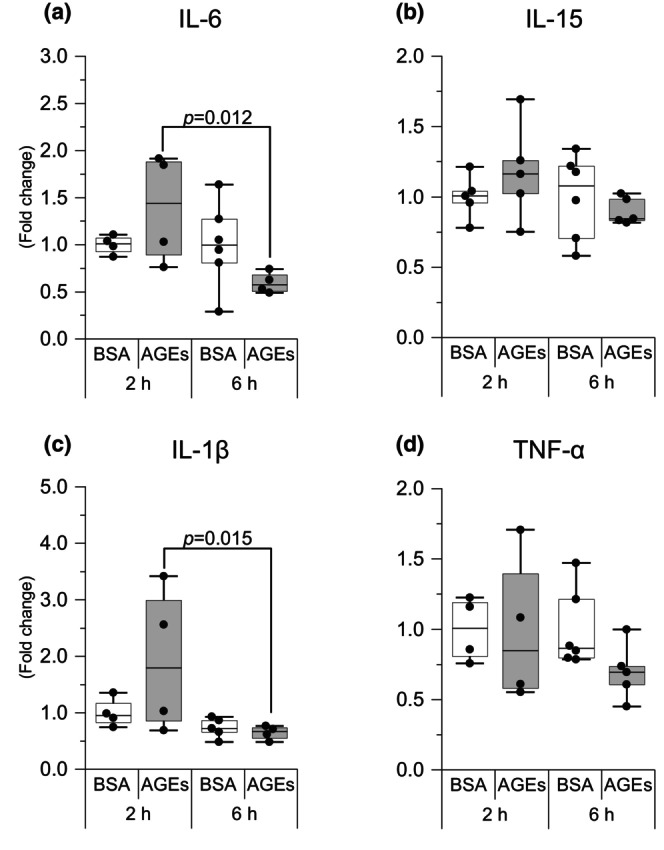
AGEs affect the mRNA level of inflammatory cytokine. (a) Interleukin‐6 (IL‐6). (b) Interleukin‐15 (IL‐15). (c) Interleukin‐1β (IL‐1β). (d) Tumor necrosis factor α (TNF‐α). Data are shown as box plots. Individual data points are indicated on the graph. Statistical significance is analyzed using two‐way ANOVA with simple effects tests. *n* = 4–6/group.

We subsequently examined changes in inflammatory cytokine expression. Six hour AGEs stimulation decreased IL‐6 expression levels compared with 2‐h AGEs stimulation (*p* = 0.012, *g* = 1.90; Figure 7a). Six‐hour AGEs stimulation decreased IL‐1β expression levels compared with 2 h AGEs stimulation (*p* = 0.015, *g* = 1.40; Figure 7d). AGEs had no effect on IL‐15 and TNFα expressions (Figure 7b,e). These results suggest that AGEs alter the production of inflammatory cytokines.

## 4 DISCUSSION

**FIGURE 8:**
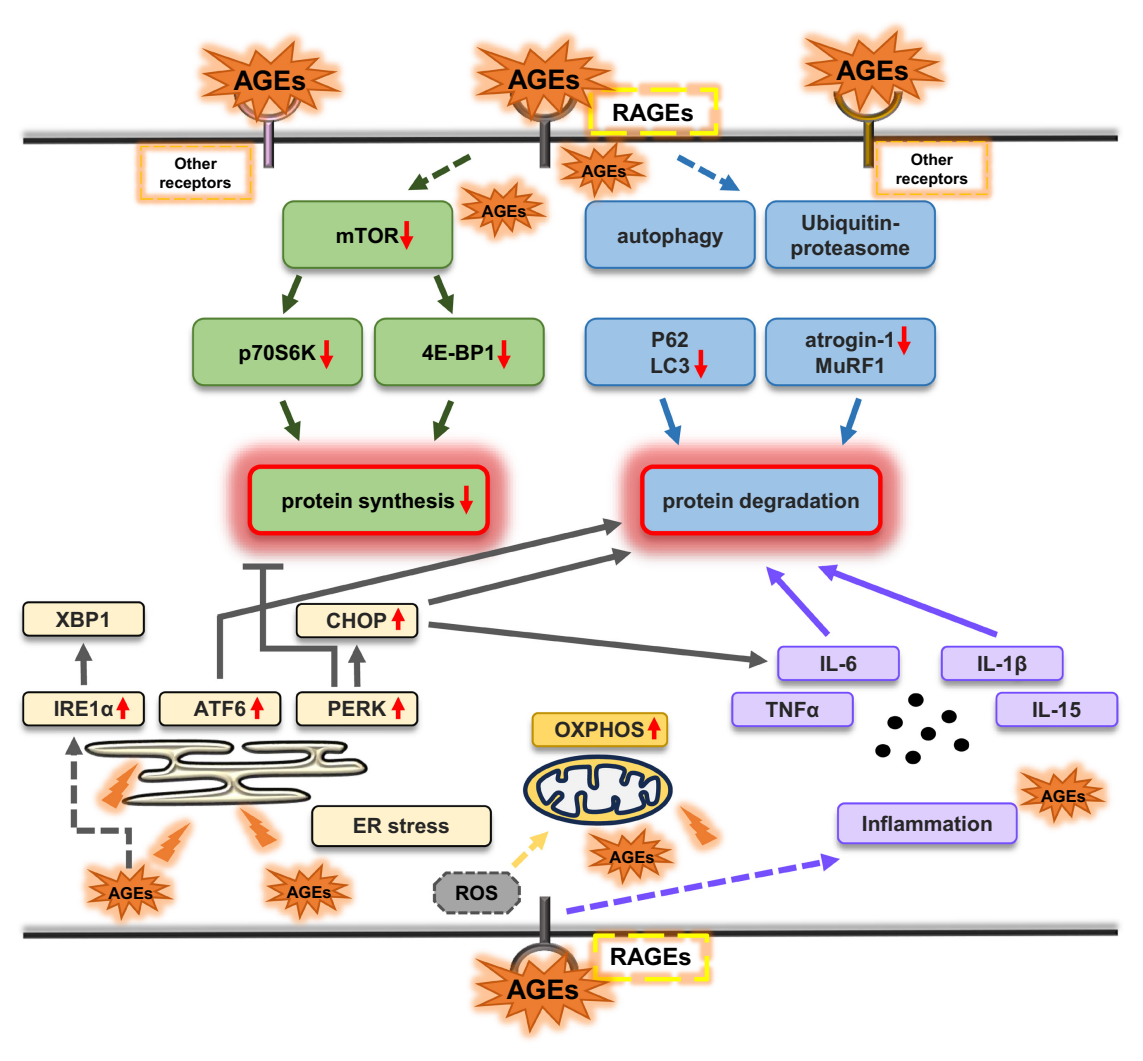
Graphic summary of the acute effects of AGEs on skeletal muscle proteostasis.

Several previous studies have shown that systemic exposure to dietary AGEs affects the skeletal muscle; however, the possibility that these effects are mediated by other organs has not been excluded. In this study, we used isolated skeletal muscle from mice for AGEs stimulation. This eliminates the influence of other factors in the organism and allows observing the direct effect of AGEs on the molecules in skeletal muscle tissues. The results obtained showed that in isolated EDL acutely (≤6 h) stimulated with AGEs, protein synthesis and the mTOR signaling pathway were suppressed, and normal protein degradation pathways (autophagy and the ubiquitin–proteasome system) were disrupted. Furthermore, ER stress was induced by modulating ATF 6 expression, PERK phosphorylation, and CHOP expression, accompanied by changes in mitochondria proteins and inflammatory cytokines (Figure 8).

In skeletal muscle cells, RAGE is a transmembrane receptor located predominantly on the cell membrane. Although RAGE has been reported to localize mitochondria and nucleus, physiological relevance of their RAGE is unknown (Riuzzi et al., 2018). It can bind to various ligands, including AGEs. AGEs bind to RAGE on the cell membrane, activating downstream molecules such as Erk1/2, p38MAPK, JNK, and others to actives intracellular signaling pathway (Khalid et al., 2022; Muthyalaiah et al., 2021). Therefore, we examined the phosphorylation of the molecules to verify the activation of the AGEs‐RAGE axis. The results revealed that AGEs stimulation for 6 h significantly increased the phosphorylation of each molecule (Figure 3). These results suggest that AGEs affect muscle cells through the AGEs‐RAGE axis.

Muscle atrophy increases with age, thereby leading to higher mortality rates and affecting metabolic status (Paez et al., 2023); AGEs that accumulated with age have been proven to diminish skeletal muscle mass (Egawa et al., 2017, Pinto et al., 2018). The mTOR signaling pathway regulates translation initiation by activating p70S6K (Wang et al., 2001). In this study, we showed that p70S6K phosphorylation is inhibited by AGEs (Figure 2c), which is consistent with our previously reported results in vivo (Egawa et al., 2017; Egawa et al., 2018). 4E‐BP1 dissociates from eukaryotic translation initiation factor 4E through mTOR‐mediated phosphorylation (Gingras et al., 2001), thereby facilitating the recruitment of ribosomal machinery and cap‐dependent mRNA translation. We observed that 4E‐BP1 phosphorylation was also inhibited by AGEs (Figure 2d). These findings suggest that exposure to AGEs for at least 6 h to the skeletal muscle inhibits protein synthesis via the mTOR signaling pathway. This is also consistent with the decrease in puromycin‐labeled protein level observed using the SUnSET method.

Protein degradation is a mechanism to remove cytoplasmic components, such as abnormal proteins, and is also one of the significant ways to maintain skeletal muscle mass (Masiero et al., 2009). A previous study showed that AGEs activate autophagy and the ubiquitin–proteasome system in diabetic skeletal muscle and prolonged (48 h) exposure to cultured skeletal muscle cells (Chiu et al., 2016). Interestingly, our results showed a decrease in LC3II/LC3I and atrogin‐1 at 6 h of AGEs stimulation (Figure 4c,e), suggesting that AGEs acutely (≤6 h) inhibit intracellular protein degradation. The chronic inhibition of basal autophagy leads to the onset of muscle atrophy, decreased muscle power generation capacity, impaired cell organelle and protein accumulation, and ultimately early death (Hussain et al., 2021). Furthermore, autophagy is necessary for satellite cell activation, which is a significant mechanism for muscle regeneration (Fiacco et al., 2016). The obstacle of autophagy can induce contractile protein oxidation, thereby causing muscle weakness (Carnio et al., 2014). Therefore, it is suggested that AGEs may disrupt proteostasis and thereby induce skeletal muscle dysfunctions or have a biphasic effect, thereby inhibiting protein degradation for short periods and enhancing it for longer periods.

The ER stress is a potential factor for muscle mass wasting (Zheng et al., 2023). PERK and ATF6 are activated as sensing proteins of ER stress (Hetz, 2012). PERK has been reported to be essential for the maintenance of skeletal muscle mass in mice with cancer cachexia (Gallot et al., 2019). In this study, AGEs upregulated PERK phosphorylation in the skeletal muscle at 6 h (Figure 5e) and upregulated ATF6 expression at 2 h (Figure 5b), indicating acute ER stress induction by AGEs, which is consistent with the results of a previous study (Egawa et al., 2020). When PERK is activated, it phosphorylates the α‐subunit of eukaryotic translation initiation factor 2, leading to its inactivation. This rapid reduction in translation initiation inhibits overall protein synthesis (Elvira et al., 2020). When activated under ER stress conditions, ATF6 translocates to the nucleus and induces the expression of genes involved in protein folding, trafficking, and degradation to restore ER homeostasis (Glembotski et al., 2020). CHOP, as a significant downstream molecule of the ER stress pathway, induces apoptosis (Kim et al., 2022). CHOP can induce the expression of E3 ubiquitin ligases, such as atrogin‐1 and MuRF1, leading to increased protein degradation (Li et al., 2014). In addition, CHOP deficiency has been shown to promote atrophy in surgically denervated muscles through macroautophagy activation (Yu et al., 2011). In our results, although 2 h of AGEs stimulation increased CHOP expression, 6 h of AGEs stimulation reduced CHOP expression (Figure 5c). This suggests that AGEs may be involved in the transient induction of CHOP expression and thereby regulating normal protein degradation, but not in the longer‐term or chronic activation of ER stress by AGEs. Although AGEs increased IRE1 Ser^724^ phosphorylation at 6 h (Figure 5f), XBP‐1 s as its downstream molecule remained unchanged (Figure 5d). Thus, we speculate that the ER stress generated by AGEs stimulation is mediated by PERK rather than IRE1 and affects proteostasis through PERK and its downstream molecule, CHOP.

We observed that AGEs promote mitochondrial respiratory chain complexes II, III, and V and PGC‐1α expression at 6 h (Figure 6), suggesting that AGEs stimulation increased mitochondria proteins. This is contrary to the previous results. There are studies indicating that mice fed with an AGEs diet exhibit a reduction in the mitochondrial respiratory chain complexes I and IV activity, as well as decreased intracellular adenosine triphosphate (ATP) levels in the brain (Akhter et al., 2020). Furthermore, in the previous study, we reported that methylglyoxal, a precursor of AGEs, reduces exercise‐induced mitochondrial adaptations in mouse skeletal muscle (Egawa et al., 2022). In this regard, one study showed that 1 h of H_2_O_2_ stimulation significantly increased the expression of OXPHOS proteins, possibly because oxidative stress‐mediated reactive oxygen species (ROS) alter OXPHOS function (Noh et al., 2013). Furthermore, AMPK activation induced by ROS‐mediated ATP depletion in skeletal muscle cells promotes PGC‐1α transcription (Barbieri & Sestili, 2012). Consequently, we speculate that an increase in ROS levels due to AGEs stimulation may transiently stimulate mitochondrial biosynthesis and increase OXPHOS levels. However, sustained long‐term ROS exposure may induce mitochondrial damages. The precise mechanism by which AGEs activate mitochondrial function remains unclear and requires further investigation in future research.

Moreover, we detected several inflammatory cytokines produced by skeletal muscles because AGEs stimulate macrophages to increase various cytokines, such as IL‐6 (He et al., 2020), and because our previous studies show that chronic stimulation with AGEs promotes inflammatory cytokine expression (Egawa et al., 2020; Pinto et al., 2018). IL‐1β is a potent mediator of the inflammatory response and can induce the expression of other proinflammatory cytokines, including IL‐6, an inflammatory factor, and participates in skeletal muscle atrophy (Haddad et al., 2005). Previous studies have shown that CHOP is an inducer of IL‐6 (Hattori et al., 2003) and IL‐1β (Endo et al., 2006), suggesting that AGEs may regulate inflammatory factors via CHOP.

An important limitation of studies using isolated muscle is that external drug stimuli may not fully penetrate the internal muscle fibers. Although EDL is a muscle commonly used in isolated muscle incubation experiments, it cannot be determined from this study whether AGEs are acting on the internal myofibers. Therefore, it is important to keep in mind that the results of this study are the average of changes in muscle fibers on which AGEs have acted and those on which they have not. It would be possible to obtain clearer results by using thinner muscles, such as the flexor digitorum brevis muscle.

In this study, we used a concentration of 1 mg/mL AGEs to incubate isolated muscle tissue. This concentration is based on our previous research, which also used 1 mg/mL of AGEs to treat C2C12 muscle cells in vitro to investigate the effect on primary cilia (Suzuki et al., 2023) and cell membrane (Egawa et al., 2024). This concentration is above the physiological level (ng‐μg/mL) but is not cytotoxic (Egawa et al., 2024) and may effectively simulate cellular changes in a high‐AGEs environment. In our preliminary experiments, we found that 1 h AGEs stimulation altered inflammatory factors levels, but the changes were not significant. In another study, acute effects of AGEs have shown that the increase in reactive oxygen species intermediates in response to AGEs stimulation peaks at 6 h (Mahali et al., 2014). On this basis, the incubation times were determined to be 2 and 6 h.

## 5 CONCLUSION

Our findings showed that the short‐term (≤6 h) exposure of isolated mouse EDL muscle to AGEs activated RAGE signaling‐associated molecules, inhibited protein synthesis and related signaling pathways, and decreased protein degradation pathways, suggesting that AGEs stimulation directly act on skeletal muscle tissues and acutely affect proteostasis. Furthermore, AGE‐induced ER stress may have an impact on proteostasis. These findings provide significant evidence to elucidate the physiological effects of endogenous and dietary AGEs on the skeletal muscle.

## FUNDING INFORMATION

This research was funded by Japan Society for the Promotion of Science, grant number 18H03148, 21H03319, 22K18413, 22K19750, and 23H03283. Additional research grants were provided by the Uehara Memorial Foundation (TE), the Nakatomi Foundation (TE), the Re‐search Institute for Production Development (TE), the Kyoto University Foundation (TH), and the ISHIZUE 2023 of Kyoto University.

## CONFLICT OF INTEREST STATEMENT

The authors declare that they have no conflict of interest.

## Data Availability

The data that support the findings of this study are available from the corresponding author, upon reasonable request.
